# MicroRNA-26a-5p is a reliable biomarker in the adjuvant setting for pancreatic ductal adenocarcinoma

**DOI:** 10.1371/journal.pone.0310328

**Published:** 2024-09-17

**Authors:** Yu Takeda, Daisaku Yamada, Shogo Kobayashi, Kazuki Sasaki, Yoshifumi Iwagami, Yoshito Tomimaru, Takehiro Noda, Hidenori Takahashi, Tadafumi Asaoka, Junzo Shimizu, Yuichiro Doki, Hidetoshi Eguchi

**Affiliations:** 1 Department of Gastroenterological Surgery, Graduate School of Medicine, Osaka University, Suita, Osaka, Japan; 2 Department of Surgery, Osaka Police Hospital, Osaka, Japan; 3 Department of Surgery, Toyonaka Municipal Hospital, Toyonaka, Osaka, Japan; Penn State Health Milton S Hershey Medical Center, UNITED STATES OF AMERICA

## Abstract

Pancreatic ductal adenocarcinoma (PDAC) has a high recurrence rate even after radical resection because of subclinical tumors. To manage them, a reliable biomarker that can indicate the presence of subclinical tumors and predict their chemosensitivity is required. This study aimed to identify a miRNA as a biomarker that can be used to individualize postoperative adjuvant chemotherapy using postoperative peripheral blood samples. Integrating miRNA microarray data from the blood of 18 patients with PDAC and the in vitro results regarding the phenotypes of chemoresistant PDAC cells, a candidate miRNA was identified. The relationships between candidate miRNA expression and chemosensitivity were examined *in vitro* and in clinical samples from other cohorts of 33 patients with recurrence. Comprehensive analyses of blood samples detected 5 candidate miRNAs. Of these, miR-26a-5p was considered a candidate biomarker of chemosensitive phenotypes. In validation experiments, chemosensitivity was inversely correlated with miR-26a-5p expression *in vitro*. Moreover, the ability of miR-26a-5p to predict chemosensitivity was clinically evaluated using blood samples. Patients with high miR-26a-5p expression in the blood after radical resection exhibited a significantly longer survival time after recurrence. Thus, we concluded that miR-26a-5p is a potentially useful biomarker for managing patients with PDAC, especially those undergoing adjuvant chemotherapy.

## Introduction

Pancreatic ductal adenocarcinoma (PDAC) is a lethal disease with a poor prognosis. Surgery for patients with PDAC is the only possible cure; however, most patients develop recurrence at an extremely high rate of 80–90%, even after radical resection at an early stage [1]. Tumor cells that are undetectable on imaging are potential seeds of recurrence; nevertheless, the current PDAC evaluation system typically comprises imaging investigations, and the detection of all tumor cells in the patient’s body is limited. However, these undetectable tumor cells, namely, subclinical tumors, are expected to be more valid for chemotherapy than mass-forming tumor sites because their cells consist of a small number of tumor cells. Postoperative adjuvant chemotherapy for subclinical tumors of PDAC has been associated with a significant decrease in the recurrence rate [2, 3]. Thus, multimodal therapy for PDAC, including surgery and chemotherapy, is a reasonable strategy. Surgery removes a conspicuous (visible) tumor site, and postoperative chemotherapy is used to treat subclinical tumors. Thus, postoperative adjuvant chemotherapy could be the optimal treatment; however, a significant issue remains as there is no established biomarker indicating subclinical tumors that should be targeted by adjuvant chemotherapy. Therefore, current adjuvant chemotherapy involves the uniform administration of adjuvant chemotherapy for uniform terms after tumor resection in all patients, regardless of subclinical tumor presence/phenotype. This presents a challenge because some patients undergo prolonged standard adjuvant chemotherapy, or a multidrug regimen is suitable for certain patients, and several patients may not require adjuvant chemotherapy. If a biomarker indicating the presence of subclinical tumors is available, it will be of tremendous benefit to decision-making regarding the discontinuation, continuation, or alteration of the chemotherapy being administered. Moreover, if the biomarker also indicates the phenotypes of subclinical tumors that are sensitive to chemotherapy, it would be immensely advantageous in optimizing treatment. At present, carbohydrate antigen 19–9 (CA19-9) is a long-standing PDAC-associated biomarker that monitors the response to treatment [4]; however, its value does not indicate tumor phenotypes, and a new, reliable biomarker that can address these shortcomings needs to be established.

Several biomarkers established from blood-based liquid biopsies have demonstrated potential for PDAC diagnosis and staging [5, 6]. MicroRNAs (miRNAs) are endogenous 18–25 nucleotide, highly conserved, non-coding, single-stranded RNAs that are synthesized by cancer cells in the blood [7]. Synthesized miRNAs are stable in blood and can be detected in peripheral blood; thus, investigating miRNA expression in blood is a type of liquid biopsy. Several miRNA molecules have been reported as useful biomarkers for the early detection of tumor development [7–11]. Moreover, miRNA plays a role in the binding of transcriptional suppressors to target mRNAs with incomplete homology and influence biological processes, such as development, cell proliferation, apoptosis, and chemoresistance [12, 13]. Thus, miRNA expression in peripheral blood is potentially useful as a biomarker not only for the presence of subclinical tumors, but also for chemosensitive tumor phenotypes.

The timing of blood collection is the most significant issue in the evaluation of blood miRNAs derived from subclinical tumors. The blood samples obtained from patients with primary PDAC may contain miRNAs derived from both primary and subclinical tumors, and they may differ. Therefore, we focused on blood samples obtained from patients who had undergone radical resection and investigated blood miRNAs derived from subclinical tumors. This study aimed to identify biomarkers that can be used to individualize postoperative adjuvant chemotherapy using postoperative peripheral blood samples. To identify miRNAs secreted by subclinical tumors, we assessed cases with no recurrence without receiving adjuvant chemotherapy, assuming that these cases had no subclinical tumors. In contrast, cases with recurrence, regardless of whether they received adjuvant chemotherapy, were assumed to have subclinical tumors. We identified miRNAs in the blood that were significantly elevated in these cases having subclinical tumors. Subsequently, among the recurrent cases, we identified miRNAs that were elevated in cases where recurrence occurred during or immediately after adjuvant chemotherapy, suggesting resistance to the treatment. This allowed us to identify miRNAs that could serve as blood biomarkers associated with the presence of subclinical tumors and their sensitivity to adjuvant chemotherapy.

## Materials and methods

### Clinical samples

Prior to study inclusion, all patients provided written informed consent for the use of the research results. The study protocol was approved by the Human Ethics Review Committee of the Graduate School of Medicine, Osaka University (approval number: 22208), and it adhered to the guidelines of the Declaration of Helsinki. In this study, patients who underwent surgery between April 2012 and December 2014 and had blood samples taken at nearly the same postoperative timing were targeted. The dates when blood samples and resected specimens were accessed for research purposes in 01/09/2022. These cases were included with prior written informed consent obtained for the possibility of conducting a retrospective study using the information and the potential use of preserved samples. Notification of the use of these cases for this study was made via opt-out on our department’s website.

### Blood samples

Before the initiation of adjuvant chemotherapy, postoperative blood samples were obtained from patients who had undergone pancreatectomy with R0 resection for PDAC at Osaka University Hospital. A total of 51 peripheral blood samples were examined for two purposes: investigation and validation. Blood samples were immediately centrifuged at 3000 rpm at 4°C for 5 min, and the supernatant was collected and stored at –80°C. These blood samples were collected around 30 days after surgery (22–53 days).

To investigate candidate miRNAs, microarray analyses of miRNA expression in 18 blood samples (Table 1) was performed using the Toray microRNA microarray system (Toray Industries, Tokyo, Japan). Labeled RNAs were hybridized onto 3D-Gene^®^ Human miRNA Oligo chips (v.17.0; Toray Industries, Tokyo, Japan). Fluorescent signals were scanned using a 3D-Gene^®^ Scanner (Toray Industries, Japan) and analyzed using 3D-Gene^®^ Extraction software (Toray Industries, Japan). Using these data, two different comparisons among 18 patients were performed. In the comparisons, we assumed that the change in expression was > 1.5-fold, and statistical significance was set at p < 0.05.

**Table 1:** Characteristics of the patients and blood samples used to investigate candidate miRNAs (n = 18).

Age (years)	67 ± 8
Sex (male / female)	13 / 5
Tumor location (head / body or tail)	9 / 9
Preoperative treatment (yes / no)	12 / 6
pT Stage (Tis,1,2 / 3,4) UICC ver.7	3 / 15
pN Stage (0 / 1,2) UICC ver.7	11 / 7
Postoperative CEA (ng/mL)	2.9 ± 2.3
Postoperative CA19-9 (U/mL)	69 ± 88
Postoperative DUPAN-2 (U/mL)	202 ± 601
Adjuvant chemotherapy (yes / no)	15 / 3
Tumor recurrence (yes / no)	11 / 7

Data are expressed as the number of patients or mean ± standard deviation, as indicated.

UICC ver.7, Union for International Cancer Control TNM classification 7^th^ edition; CEA, carcinoembryonic antigen; CA19-9, carbohydrate antigen 19–9; DUPAN-2, pancreatic cancer-associated antigen-2

To validate the candidate gene’s predictive ability for chemo-susceptibility, we used 33 entirely different blood samples from patients who had developed postoperative recurrence. (Table 2).

**Table 2:** Characteristics of the patients and blood samples used to validate miRNA predictive ability (n = 33).

Age (years)	72 ± 9
Sex (male / female)	22 / 11
Postoperative CEA (ng/mL)	1.9 ± 1.2
Postoperative CA19-9 (U/mL)	79 ± 161
Postoperative DUPAN-2 (U/mL)	74 ± 157
Preoperative treatment (yes / no)	24 / 9
Completion of adjuvant chemotherapy (yes / no)	13 / 20
Tumor location (head / body or tail)	18 / 15
Tumor size (mm)	26 ± 12
pT Stage (Tis,1,2 / 3,4) UICC ver.7	5 / 28
pN Stage (0 / 1,2) UICC ver.7	17 / 16
Recurrence sites (local / distant metastasis)	5 / 28

Data are expressed as the number of patients or mean ± standard deviation, as indicated.

UICC ver.7, Union for International Cancer Control TNM classification 7^th^ edition; CEA, carcinoembryonic antigen; CA19-9, carbohydrate antigen 19–9; DUPAN-2, pancreatic cancer-associated antigen-2

### Resected specimen

The resected specimens were immediately fixed in 10% formalin for 48 hours. Thereafter, they were embedded in paraffin and sectioned into 4-μm slices for further evaluation, as described previously. A proportion of the slides was routinely stained with hematoxylin and eosin for pathological evaluation by certified pathologists at our institution. The remaining slides were examined using immunohistochemistry. Of these slides, the available 28 resected specimens from the patients corresponding to the 33 patients whose blood samples were evaluated for the validation analysis were evaluated for the target protein expression of the candidate gene.

### Conventional PDAC cell lines, gemcitabine resistant cell lines, and the cell culture

We used three human PDAC cell lines (Panc1, MiaPaCa2, and BxPC3): Panc1 (NCBI_Iran Cat# C556, RRID: CVCL_0480) was obtained from the American Type Culture Collection (VA, USA), and MiaPaCa2 (NCBI_Iran Cat# C459, RRID: CVCL_0428) and BxPC3 (RRID: CVCL_0186) were obtained from the Japan Cancer Resource Bank (Tokyo, Japan). The cells were cultured as previously described [14]. Briefly, the cells were cultured in Dulbecco’s modified Eagle’s medium (DMEM), supplemented with 10% heat-inactivated FBS, and incubated at 37°C in a humidified incubator with 5% CO_2_.

We used stable gemcitabine (GEM)-resistant (GR) cell clones established from Panc1 and MiaPaCa2 cells and named them Panc1-GR1, -GR2, -GR4, -GR7, and MiaPaCa2-GR10. GEM-resistant cells were established by exposure to gradually increasing concentrations of the drug for 2 months, as previously described [15–17]. GEM was purchased from Eli Lilly Pharmaceuticals (Indianapolis, IN, USA). These GEM-resistant cells showed cross-resistance to 5-FU treatment.

### Microarray assay with messenger RNA and Gene Set Enrichment Analysis (GSEA)

Messenger RNA (mRNA) microarray analysis was performed by Toray Industries (Tokyo, Japan) using the TORAY 3D‐Gene^®^ platform. Purified RNA molecules were obtained from Panc1-Pt, Panc1-GR1, -GR2, -GR4, and-GR7 cells. To compare chemosensitive and chemoresistant cells, we performed GSEA (version 4.2.2; Broad Institute, MA, USA,). Biologically defined gene sets were obtained from the Molecular Signatures Database (RRID: SCR_016863, v7.2; http://software.broadinstitute.org/gsea/msigdb/index.jsp).

### TargetScan analysis

The targets of candidate miRNA genes were explored using TargetScanHuman (https://www.targetscan.org). Each of the candidate miRNAs extracted from the miRNA microarray assay of clinical blood samples was identified using TargetScan and matched with the genes involved in the GSEA-detected gene sets.

### Quantitative real-time polymerase chain reaction (qRT-PCR)

Total RNA was isolated from cell lines and blood samples using the miRNeasy Mini Kit (Qiagen, Hilden, Germany) and miRNeasy Serum/Plasma Advanced Kit (Qiagen, Hilden, Germany), respectively. To evaluate candidate gene expressions *in vitro*, miRNAs were purified from the cell culture supernatant to mimic the blood-sample environment.

To evaluate mRNA expression, qRT-PCR was performed as previously described [18]. Complementary DNA was synthesized using a Reverse Transcription System (Promega, WI, USA). Amplification products were quantified using THUNDERBIRD^®^ SYBR qPCR Mix (TOYOBO, Osaka, Japan), and target gene expression levels were normalized to β-actin expression levels. The PCR primer sequences used are listed in Table 3. Data were analyzed using the comparative C_T_ method.

**Table 3:** PCR primer sequences.

Oligonucleotides	Base sequences
Beta-actin forward	5′- TTAAGGAGAAGCTGTGCTACG -3′
Beta-actin reverse	5′- GAGGGGCCATCCACAGTCTTC -3′
Integrin alpha 5 (ITGA5) forward	5′- CCTGCTGTCCATGTCTATGA -3′
Integrin alpha 5 (ITGA5) reverse	5′- TGGTCACATATAGGAGCTGCTGAC -3′
Integrin alpha 6 (ITGA6) forward	5′- CGAAACCAAGGTTCTGAGCCCA -3′
Integrin alpha 6 (ITGA6) reverse	5′- CTTGGATCTCCACTGAGGCAGT-3′
Integrin beta 8 (ITGB8) forward	5′- TGTGAAGCAGGCAGATGCCAATG -3′
Integrin beta 8 (ITGB8) reverse	5′- GCCTCTTCCACTGCACACTTGG- 3′

For miRNA, reverse transcription was performed using the mir-X miRNA First Strand Synthesis Kit (TaKaRa Bio, Shiga, Japan). For qRT-PCR, we used the mir-X miRNA qRT-PCR TB Green Kit (TaKaRa Bio, Shiga, Japan) and ViiA 7 software (Thermo Fisher Scientific, MA, USA). The sequences of the hsa-miR-26a-5p, miR-103a-3p, miR-191-5p, and miR-423-3p primers were designed based on miRBase (RRID: SCR_003152, http://www.mirbase.org). Target miRNA expression in PDAC cell lines was normalized relative to U6 expression, which was used as an internal control. However, no endogenous miRNA control for normalizing miRNA levels in blood samples has been established [19]. In this study, miRNA expression levels in blood samples were normalized relative to the average expressions of the three endogenous miRNAs (miR-103a-3p, miR-191-5p, and miR-423-3p, which were confirmed to exist abundantly and stably in blood samples and were acceptable as favorable normalizers in QIAGEN’s protocol) as internal controls, as described previously [20–25].

### Growth inhibitory assay

Growth inhibition was assessed using the MTT (Sigma-Aldrich, MO, USA) assay, as described previously [26]. Briefly, cells were incubated for 72 hours at several GEM or 5-FU concentrations. After re-incubation for 4 hours with the MTT solution, plate absorbance was measured and the results are expressed as a percentage of absorbance relative to that of untreated controls.

### Invasion assays

Invasion assays were performed using Matrigel-loaded invasion chambers, according to the manufacturer’s instructions (Biocoat^TM^ Matrigel Invasion Chamber; Collaborative Biomedical Products; Corning, NY, USA), as described previously [27].

### Proliferation assay

Cell viability was assessed using the Cell Counting Kit-8 (CCK-8; Dozindo Laboratories, Kumamoto, Japan), as previously described [28]. Each cell line was seeded onto a 96-well plate (2.5 × 10^3^ cells/well) and incubated for 24 hours. At 0, 24, 48, and 72 hours after incubation, CCK-8 solution (10 μL/200 μL of cell suspension) was added to each well. The absorbance was measured using a microplate reader. The results are expressed as the ratio of the absorbance with CCK-8 exposure relative to that at 0 hours of exposure.

### Transfection

Cells were transfected with 10 nmol/L mirVana miRNA inhibitor (anti-miR-26a-5p) or non-targeting negative control (miR-NC) oligonucleotides (Ambion, Austin, TX, USA) using Lipofectamine RNAiMAX (Invitrogen, Carlsbad, CA, USA), according to the manufacturer’s protocol.

### Immunocytochemical staining

Immunocytochemistry was performed as previously described [29]. Briefly, cells were fixed with 4% paraformaldehyde, and proteins were detected using integrin alpha 5 (ITGA5)-specific antibodies (anti-ITGA5 rabbit monoclonal antibody; Abcam, Cambridge, UK). After incubation with the corresponding secondary antibodies (Cell Signaling Technology, MA, US) in the dark, the cells were mounted using 4′,6-diamidino-2-phenylindole (Dozindo Laboratories, Kumamoto, Japan) to visualize the nuclei. All images were captured using a fluorescence microscope (BZ-X700; Keyence, Osaka, Japan).

### Immunohistochemistry

Immunohistochemistry was performed to detect ITGA5 expression, as previously described [30]. Briefly, paraformaldehyde-fixed, paraffin-embedded pancreatic tissue sections were deparaffinized, hydrated, and incubated overnight at 4°C with an anti-ITGA5 antibody as the primary antibody. Bound antibodies were detected using biotin-conjugated secondary antibodies and diaminobenzidine (Vector Laboratories, Burlingame, CA, USA) as a substrate, and the tissue slices were counterstained with hematoxylin. Because ITGA gene set was identified from PDAC cancer-cell analysis, cases in which ITGA5 was stained in cancer cells (not stromal cells) were defined positive.

### Statistical analysis

Continuous variables are expressed as the mean ± standard deviation. Categorical variables were compared using the chi-square or Fisher’s exact test, as appropriate, and continuous variables were compared using the student’s t-test. The survival time after recurrence (SAR) and overall survival time (OS) were analyzed using the Kaplan–Meier method, and differences between survival curves were compared using the Wilcoxon test. To evaluate the risks associated with the prognostic variables, we performed univariate analyses using a Cox model and determined the hazard ratios and 95% confidence intervals. Receiver operating characteristic curve analysis was conducted using the Youden method for cut-off point analysis. Statistical analyses were performed using JMP software (version 16.0; SAS Institute Inc., Cary, NC, USA) (Statistical Analysis System, RRID: SCR_008567).

## Results

### Identification of candidate biomarkers using a comprehensive analysis of postoperative blood samples from patients with PDAC

Postoperative peripheral blood samples were obtained from 18 patients with PDAC who had undergone radical resection. We assumed that the patients could be divided into four groups based on whether adjuvant chemotherapy (AC) was administered (AC +/–) and whether recurrence (Rec) developed (Rec +/–). Patients with R0 resection were highly recommended to receive adjuvant chemotherapy (GEM or S-1, which is a prodrug of 5-FU), but in reality, several patients did not receive it. Cases were classified as Rec (–) if they did not show recurrence for at least 3 years after surgery. Whole miRNA expression in the patients’ blood was assessed. To detect candidate genes indicating the presence of subclinical tumors, miRNA expression from the microarray data was compared using two different methods. The expression of miRNA was compared between Group (AC-, Rec-, n = 3) and Group (Rec+, n = 11) using the blood samples of 14 patients. From this comparison, 40 candidate miRNAs were detected, potentially indicating the presence of subclinical tumors and demonstrating a > 1.5-fold increase in the change in Group (Rec+).

**Fig 1:**
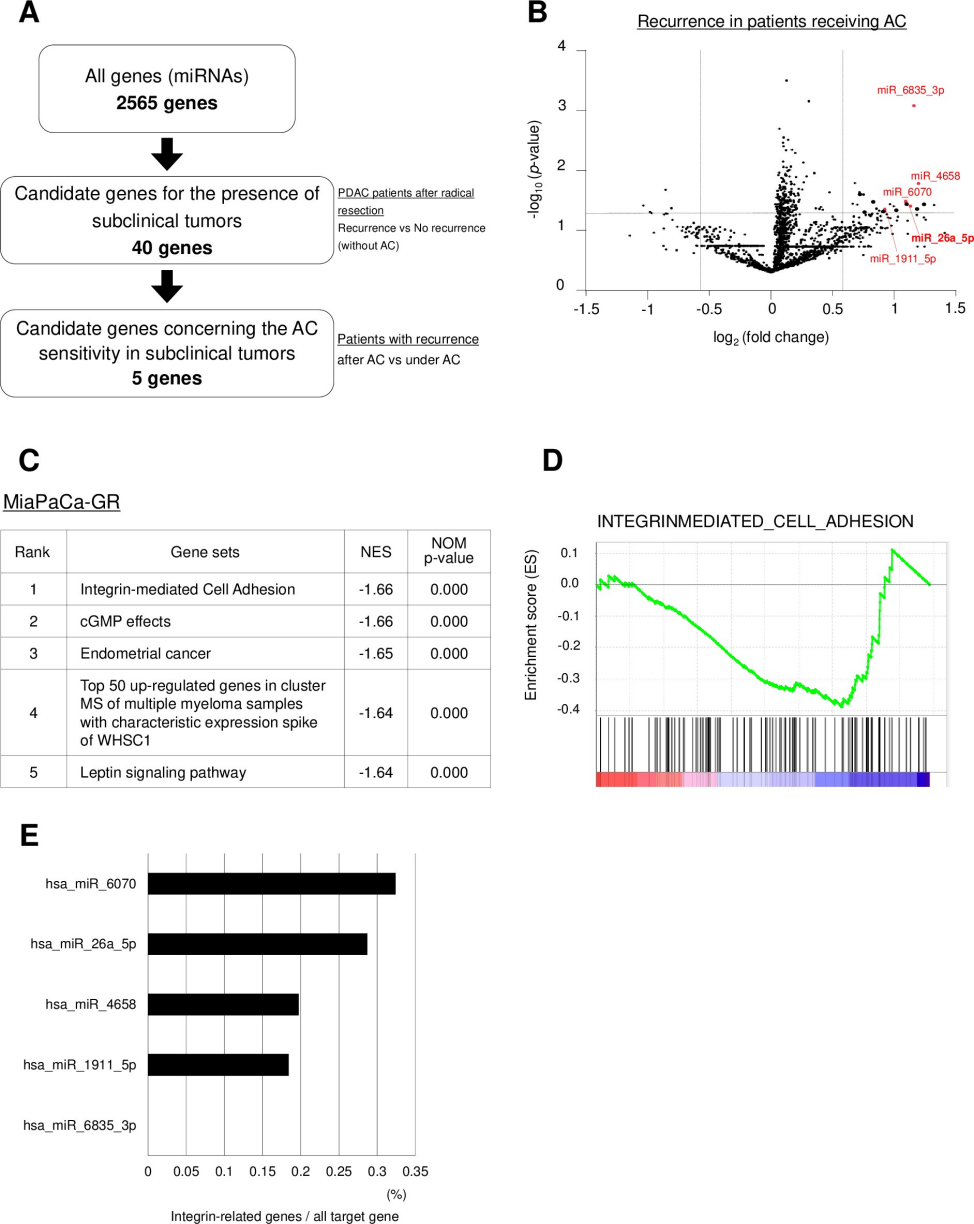
Selection of candidate miRNAs and search for target genes. **(A)** Flowchart of candidate miRNA selection. Microarray results were compared between patients with and without subclinical tumors or recurrence under or after adjuvant chemotherapy, and candidate miRNAs were isolated. **(B)** Volcano plots for candidate miRNAs in patients with recurrence during AC. (**C)(D)** GSEA comparing Panc1-GR and Panc1-Pt cells. GSEA-extracted representative gene sets enriched in these cells are shown. (**E)** List showing the ranking of candidate miRNAs from the above 5 miRNAs according to the percentage of genes related to the integrin-mediated cell adhesion pathway retrieved by TargetScan.

To isolate the candidate genes for the chemosensitive subclinical-tumor phenotypes, patients in Group (AC+, Rec+) were divided based on chemo-susceptibility to adjuvant chemotherapy and compared. We compared patients with subclinical tumors that were potentially sensitive to adjuvant chemotherapy (i.e., patients who developed recurrence at least 6 months after completing adjuvant chemotherapy, n = 5) and those with subclinical tumors that were resistant to adjuvant chemotherapy (i.e., patients who developed recurrence while undergoing adjuvant chemotherapy, n = 6). From this comparison, 28 candidate miRNAs exhibiting significant differences and > 1.5-fold changes between the groups were detected. Based on these results, 5 miRNAs were identified as candidate genes (Fig 1A and 1B).

### MiR-26a-5p, which is involved in the integrin-mediated cell adhesion pathway, is a candidate biomarker that suggests the presence of chemosensitive subclinical tumors

Data analysis was used to compare whole mRNA expression between the parental PDAC cells (original chemosensitive cells) and the chemo-resistant PDAC cells established from parental PDAC cells to identify chemosensitive phenotypes. We performed GSEA using mRNA microarray data. The integrin-mediated cell adhesion pathway was ranked first in the analysis (Fig 1C), and the integrin gene sets were significantly upregulated in chemo-resistant cell lines compared with those in their parental cell lines (Fig 1D), indicating that chemo-resistant cells often express integrin proteins.

Based on TargetScan analysis, the 13 candidate miRNAs were ranked according to the rate of integrin proteins in all target genes of each candidate gene (Fig 1E). Of these, we focused on miR-26a-5p (rank 2), which has been reported to be related to integrins [31], as a candidate biomarker gene that may indicate the presence of chemosensitive subclinical tumors.

### Inverse correlations of miR-26a-5p expression levels with chemo drug resistance and cell invasion

**Fig 2:**
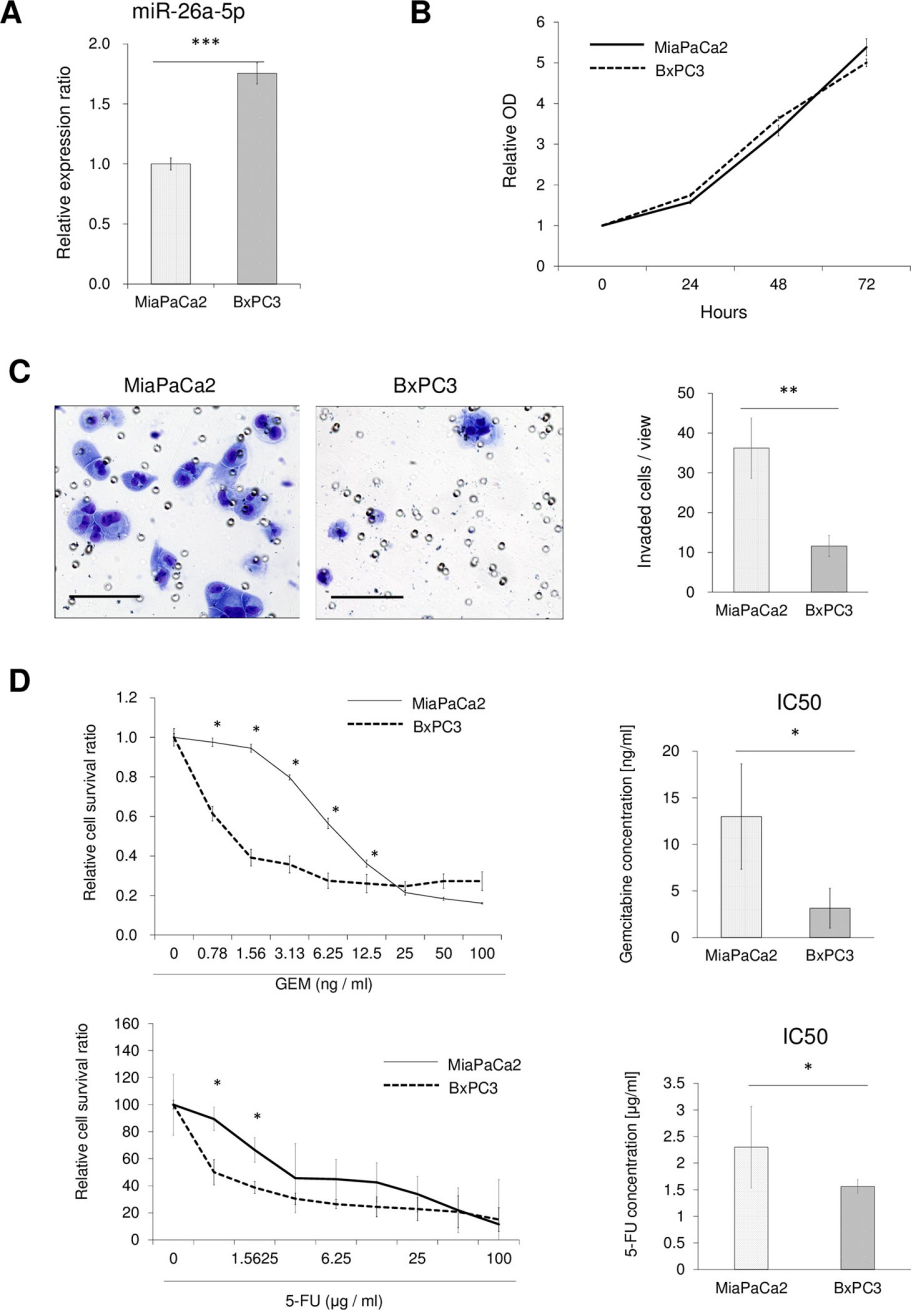
Association of miR-26a-5p expression with resistance to GEM/5-FU, cell proliferation, and cell invasive ability among PDAC cells. **(A)** MiR-26a-5p expression levels in MiaPaCa2 and BxPC3 were assessed by qRT-PCR. **(B)** The Cell Counting Kit 8 (CCK‐8) growth curves of MiaPaCa2 and BxPC3. **(C)** Invasion assays, the numbers of invaded MiaPaCa2 and BxPC3 are depicted. **(D)**Growth inhibitory assays demonstrated the relative cell survival ratio of GEM/5-FU-treated MiaPaCa2 and BxPC3, and the IC_50_ was calculated from five independent assays. Scale bar: 100 μm. *P < 0.05; **P < 0.01; ***P < 0.001.

**Fig 3:**
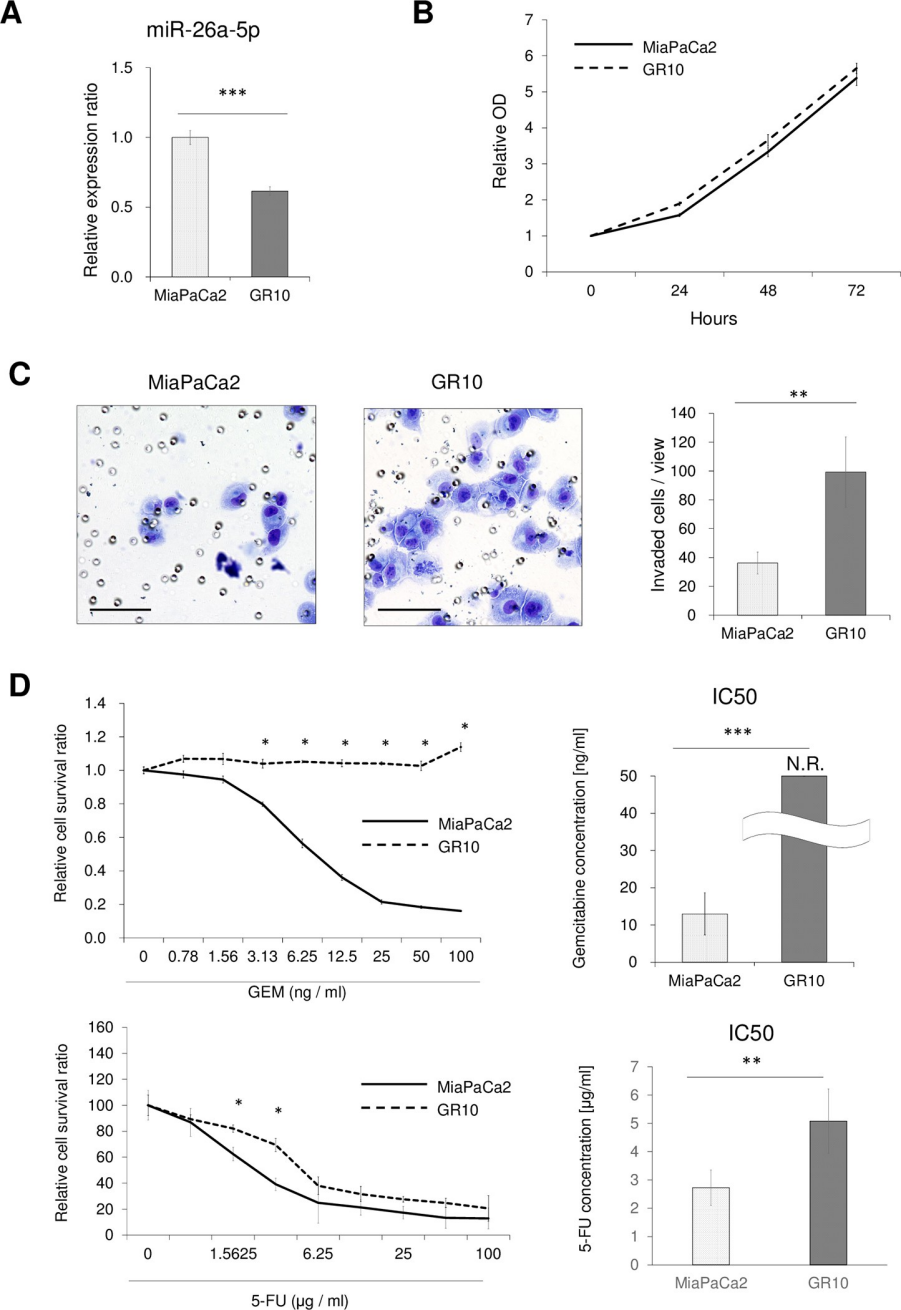
Association of miR-26a-5p expression with resistance to GEM/5-FU, cell proliferation, and cell invasive ability in chemo-sensitive/resistant cell. **(A)** MiR-26a-5p expression levels in MiaPaCa2-Pt and MiaPaca2-GR10 were assessed by qRT-PCR. **(B)** The Cell Counting Kit 8 (CCK‐8) growth curves of MiaPaCa2-Pt and MiaPaca2-GR10. **(C)** Invasion assays, the numbers of invaded MiaPaCa2-Pt and MiaPaca2-GR10 are depicted. **(D)**Growth inhibitory assays demonstrated the relative cell survival ratio of GEM/5-FU-treated MiaPaCa2-Pt and MiaPaca2-GR10, and the IC_50_ was calculated from five independent assays. For the GR cells, the IC_50_ could not be determined within the experimental concentration range, indicating that it was at least above 100 ng/ml. MiaPaCa2: MiaPaCa2-Pt, GR10: MiaPaca2-GR10, N.R.: not reached. Scale bar: 100 μm. *P < 0.05; **P < 0.01; ***P < 0.001.

To evaluate the function of miR-26a-5p *in vitro*, miR-26a-5p expression was evaluated in cell culture supernatants from two PDAC cell lines (MiaPaCa2 and BxPC3) and a GEM-resistant cell line (MiaPaCa-GR10) (Figs 2 and 3). Although no significant differences in cell proliferation ability were observed among cell lines regardless of the expression of miR-26a-5p (Figs 2B and 3B), invasion assays indicated that higher miR-26a-5p expression resulted in significantly lower invasive ability (Figs 2C and 3C). Growth inhibitory assays suggested that miR-26a-5p expression and chemosensitivity to both GEM and 5-FU were inversely correlated (Figs 2D and 3D).

**Fig 4:**
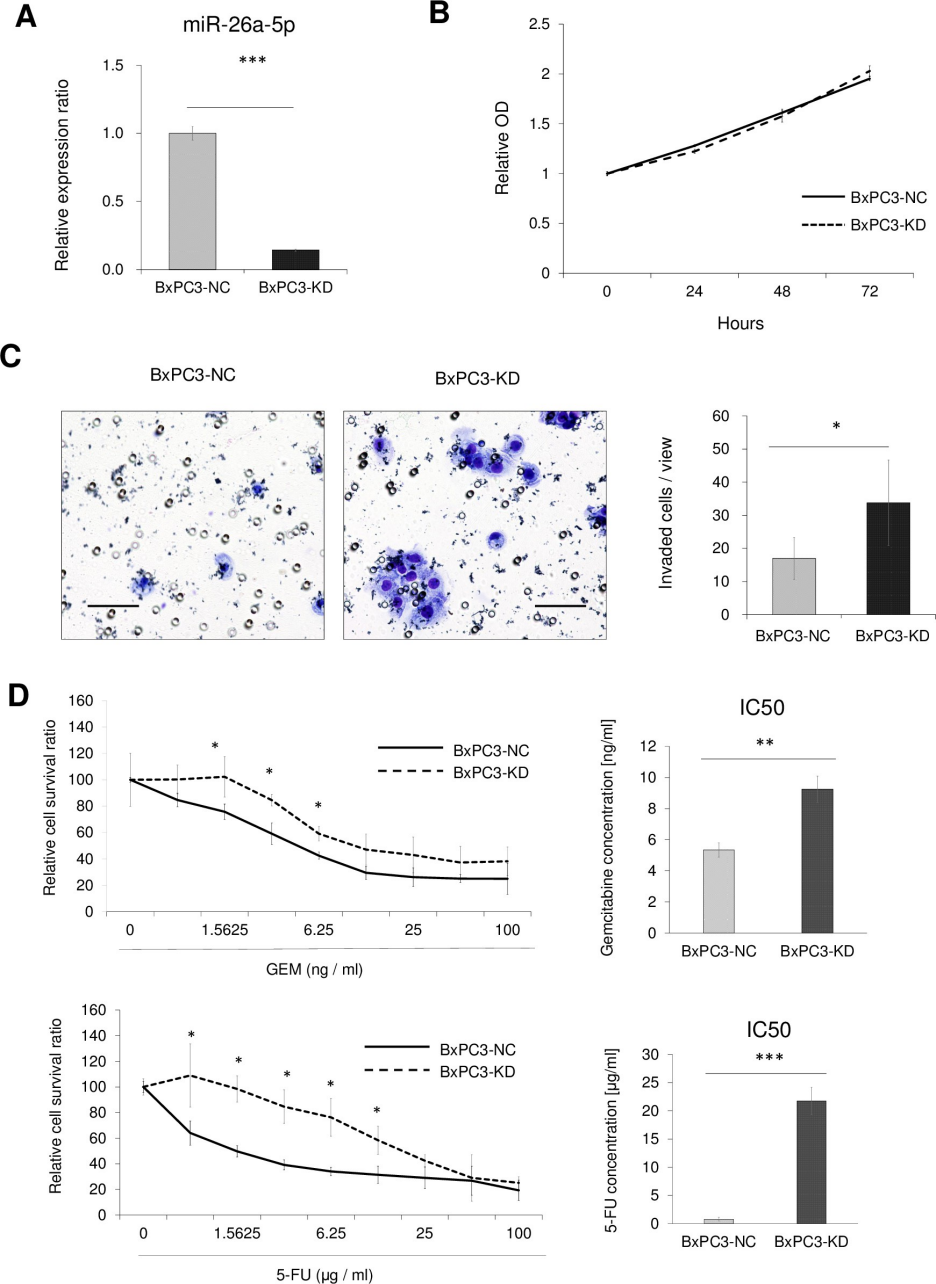
Anti-miR-26a-5p function on PDAC cells for resistance to GEM/5-FU, cell proliferation, and cell invasive ability. **(A)** MiR-26a-5p expression levels were assessed by qRT-PCR in BxPC3 cells transfected with anti-miR-26a-5p (BxPC3-KD) and negative controls (BxPC3-NC). **(B)** The Cell Counting Kit 8 (CCK‐8) growth curves. **(C)** Invasion assays. The numbers of invaded are depicted. **(D)** Growth inhibitory assays demonstrated the relative cell survival ratio of GEM/5-FU-treated BxPC3-KD and BxPC3-NC cells, and the IC_50_ was calculated from five independent assays. Scale bar: 100 μm. *P < 0.05; **P < 0.01; ***P < 0.001.

As expected, miR-26a-5p inhibition in BxPC3 cells induced chemoresistance and increased invasive ability (Fig 4). Knockdown by transfection with anti-miR-26a-5p significantly increased the half-inhibitory concentration for both GEM and 5-FU, as well as invasive ability, while a proliferative effect was not detected by miR-26a-5p inhibition (Fig 4A–4D).

### One of the most significant targets of miR-26a-5p was ITGA5

**Fig 5:**
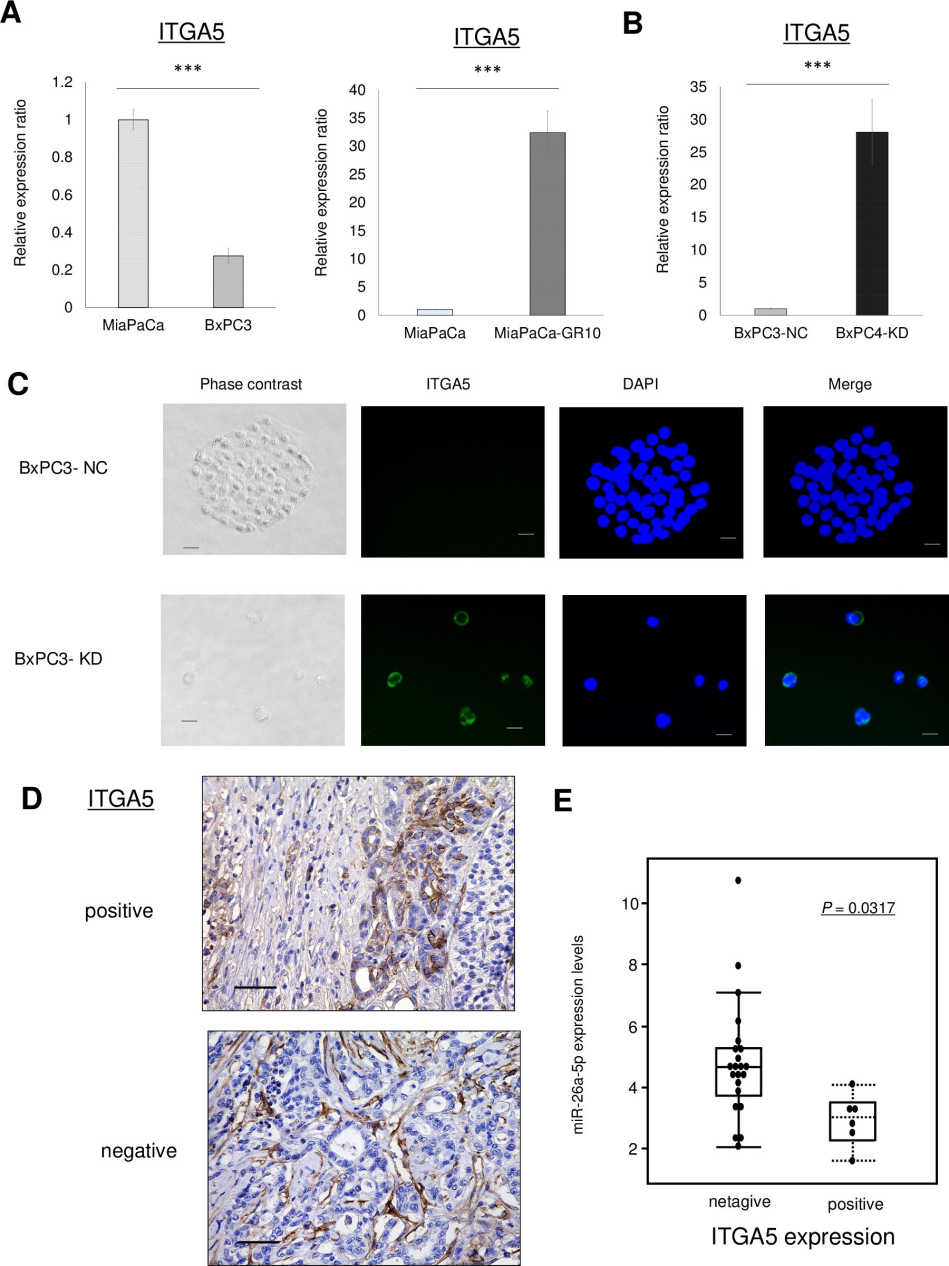
Relationship between ITGA5 and miR-26a-5p expression in PDAC cells. **(A-B)** ITGA5 expression levels were assessed by qRT-PCR in MiaPaCa2, BxPC3 **(left panel)**, MiaPaCa2-PT (MiaPaCa2), MiaPaCa2-GR10 (GR10) cells **(right panel)**, and BxPC3 cells transfected with anti-miR-26a-5p (BxPC3-KD) and negative controls (BxPC3-NC) **(B)**. **(C)** Representative images of cellular ITGA5 (green) in BxPC3-NC and BxPC3-KD cells. Immunocytochemical staining of ITGA5 (green), and the nuclei were counterstained with DAPI. **(D)** Immunohistochemical analysis of ITGA5 expression in PDAC patients’ specimens. Upper panel: ITGA5-positive expression in PDAC cells. Lower panel: ITGA5-negative expression in PDAC cells. **(E)** Box plot: The expression of miR-26a-5p in postoperative patients’ blood and the positivity of ITGA5 in the corresponding resected specimens. DAPI: 4′,6-diamidino-2-phenylindole; ITGA5: integrin alpha 5. Scale bar: 50 μm. ***P < 0.001.

Expression levels of Integrin alpha 5 (ITGA5) were significantly inversely correlated with miR-26a-5p expression (Fig 5A). Alterations in other integrin proteins showed a similar trend with changes in miR-26a-5p expression (see S1 Fig), and further investigation was conducted with ITGA5 expression due to the largest alteration observed among these integrin proteins. After confirming that knockdown of miR-26a-5p increased ITGA5 expression (Fig 5B and 5C), the relationship between ITGA5 expression in resected specimens and miR-26a-5p expression in corresponding patients’ blood samples was examined. When cases in which ITGA5 was stained in cancer cells (not stromal cells) were considered positive (Fig 5D), the expression levels of miR-26a-5p in patients with ITGA5 positivity were significantly lower than those in patients with ITGA5 negativity (Fig 5E). Based on these results, a cut-off value for miR-26a-5p in blood was established by referring to the positivity of ITGA5 in resected specimens through ROC analysis. The cut-off value of miR-26a-5p in blood samples was set at 3.4, with higher sensitivity or specificity (sensitivity: 0.667, specificity: 0.909, area under the curve: 0.697).

### In cases of postoperative recurrence, high expression of miR-26a-5p was associated with a longer duration of chemotherapy administration days

**Fig 6:**
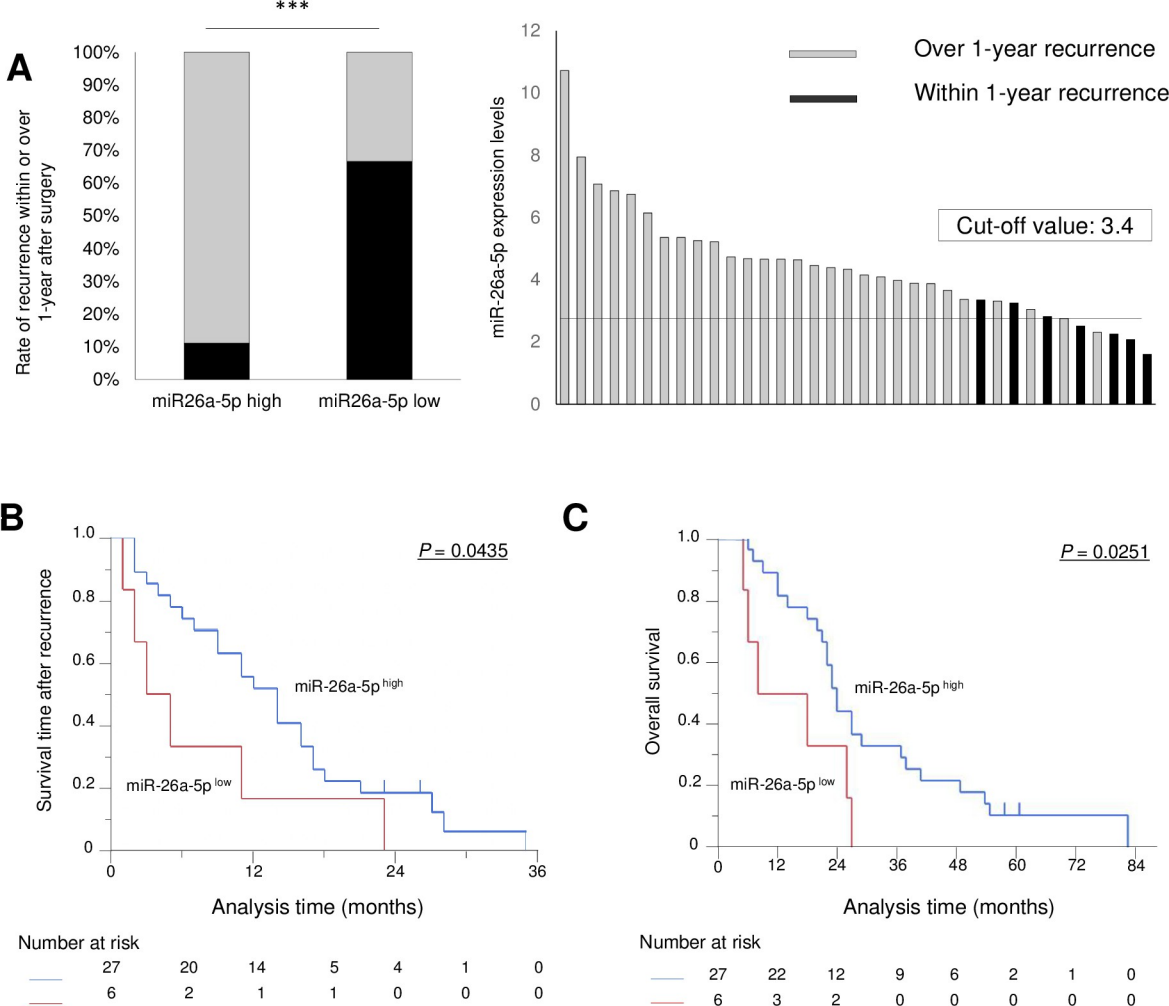
The validation of prognostic significance of miR-26a-5p expression levels in postoperative blood of PDAC patients. The validation cohort comprised 33 PDAC patients with postoperative recurrence. **(A)** The expression of miR-26a-5p and recurrence within/over 1 year after surgery. The patients were divided by a cut-off value (3.4) of miR-26a-5p expression in postoperative blood, and the rates of patients who developed recurrence within/over 1 year after surgery in each group are depicted. The bar graph shows the miR-26a-5p expression levels in each case, with patients who developed recurrence within/over 1 year after surgery represented in black. **(B)** Survival time after recurrence curves according to high or low miR-26a-5p expression. **(E)** Overall survival time curves according to high or low miR-26a-5p expression. ***P < 0.001.

Using this cut-off value, patients were divided into two groups (high/low miR-26a-5p expression, n = 27/6; Table 4). No significant differences in clinicopathological factors were noted between the high and low miR-26a-5p expression groups. Compared to the patients with low miR-26a-5p expression, those with high miR-26a-5p expression had a significantly higher rate of completion of adjuvant chemotherapy (Table 4, p = 0.0290), along with a higher rate of no recurrence within 1 year after surgery (Fig 6A). The SAR was usually significantly related to chemosensitivity because the time was significantly depended on the administration time of chemotherapy, and the SAR was significantly longer in the high miR-26a-5p expression group than in the low miR-26a-5p expression group (Fig 6B, *P* = 0.0435). Accordingly, OS was also significantly longer in the high miR-26a-5p expression group than in the low miR-26a-5p expression group (Fig 6C, *P* = 0.0251). Univariate analysis of the SAR identified only one significant factor: miR-26a-5p expression level (Table 5), suggesting that the recurrence site that developed from the subclinical tumor expressing miR-26a-5p, a chemosensitivity marker, was effectively controlled by chemotherapy.

**Table 4:** Relationship between clinicopathological factors and the miR-26a-5p expression levels in the blood samples of patients with postoperative recurrence.

	miR-26a-5p ^high^ (n = 27)	miR-26a-5p ^low^ (n = 6)	*P*-value
Age (years)	72 ± 8	72 ± 12	0.9321
Sex (male / female)	17 / 10	5 / 1	0.3384
Tumor location (head / body or tail)	13 / 14	5 / 1	0.1174
Tumor size (mm)	26 ± 12	24 ± 10	0.7494
Preoperative treatment (yes / no)	19 / 8	5 / 1	0.5190
pT Stage (Tis,1,2 / 3,4) UICC ver.7	4 / 23	1 / 5	0.9089
pN Stage (0 / 1,2) UICC ver.7	14 / 13	3 /3	0.9346
Postoperative CEA (ng/mL)	1.9 ± 1.2	2.2 ± 1.2	0.5720
Postoperative CA19-9 (U/mL)	64 ± 149	142 ± 209	0.2932
Postoperative DUPAN-2 (U/mL)	37 ± 10	81 ± 170	0.6153
Completion of adjuvant chemotherapy (yes / no)	13 / 14	0 / 6	*0*.*0290*
Recurrence sites (local / distant metastasis)	5 / 22	0 / 6	0.2525

Data are expressed as the number of patients or mean ± standard deviation, as indicated.

UICC ver.7, Union for International Cancer Control TNM classification 7^th^ edition; CEA, carcinoembryonic antigen; CA19-9, carbohydrate antigen 19–9; DUPAN-2, pancreatic cancer-associated antigen-2

**Table 5:** Univariate analyses of survival after recurrence.

		Univariate analysis	
	Parameters	Median survival time (months)	*P*-value
Age (years)	≥ 65 / < 65	11 / 14	0.5892
Sex	male / female	11 / 12	0.9848
Postoperative CA19-9 (U/mL)	> 35.4 / ≤ 35.4	11 / 11	0.1633
Postoperative DUPAN-2 (U/mL)	> 150 / ≤ 150	5 / 14	0.6338
Preoperative treatment	yes / no	9 / 16	0.0919
Tumor location	head / body or tail	9 / 14	0.1939
Tumor size (mm)	> 20 / ≤ 20	11 / 9	0.7414
pT stage (UICC ver.7)	Tis, T1, T2 / T3, T4	9 / 11	0.9201
pN stage (UICC ver.7)	N0 / N1, N2	14 / 11	0.8998
miR-26a-5p levels (2^–ΔCt^)	high / low	14 / 3	*0*.*0435*

UICC ver.7, Union for International Cancer Control TNM classification 7^th^ edition; CEA, carcinoembryonic antigen; CA19-9, carbohydrate antigen 19–9; DUPAN-2, pancreatic cancer-associated antigen-2

## Discussion

**Fig 7:**
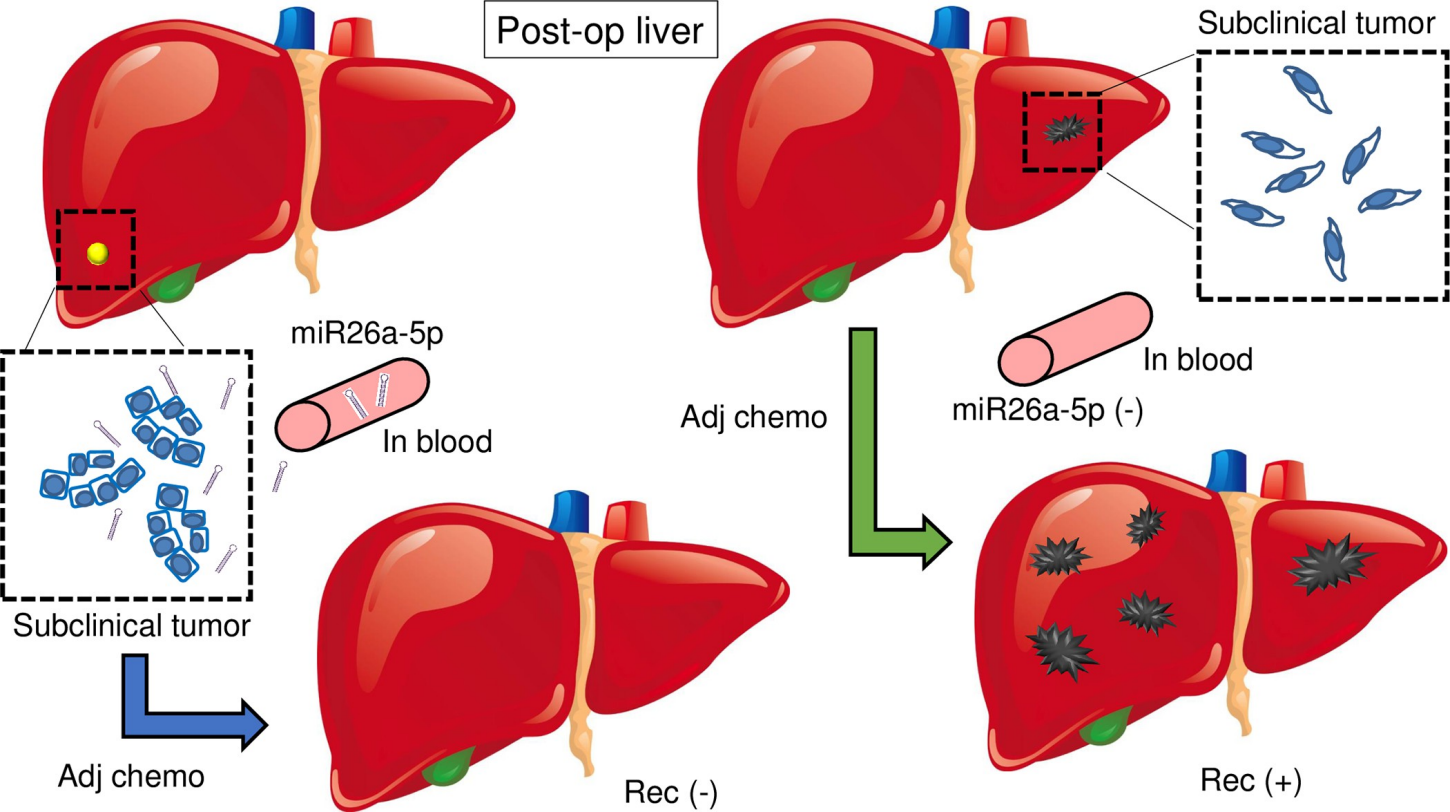
The role of microRNA-26a-5p in blood in the adjuvant setting for PDAC. Post-resection, when tumors produce miR-26a-5p, its expression in the blood increases, indicating that the tumor is more chemosensitive, which tends to suppresses recurrence with adjuvant chemotherapy. Conversely, when tumors do not produce miR-26a-5p, adjuvant chemotherapy is ineffective, leading to recurrence. Post-op liver: liver in postoperative situation, Adj chemo: adjuvant chemotherapy, Rec(-): no recurrence.

Our study demonstrated that miR-26a-5p expression in postoperative blood indicates the presence of subclinical tumors and chemo-susceptibility, and this gene could be a candidate biomarker for managing PDAC subclinical tumors (Fig 7).

We examined postoperative blood samples obtained from patients who had undergone radical resection. While blood samples from patients with PDAC have been extensively examined to identify new biomarkers, few studies have used postoperative PDAC blood samples. Therefore, we investigated the presence and properties of subclinical PDAC cells using the postoperative blood samples of patients free of conspicuous PDAC based on radical resection. Group (AC–, Rec–) comprised patients who did not have subclinical tumors and whose tumor cells were completely removed from their bodies exclusively by surgery, whereas Group (Rec+) comprised patients with subclinical tumors in their bodies after surgery. Thus, we compared these groups and regarded the more highly expressed genes in Group (Rec+) as candidate genes that indicate the presence of subclinical tumors.

To isolate the candidate genes for chemo-susceptibility, we used two completely different datasets (i.e., the comparison of clinical blood samples and cell lines) and integrated their results. Therefore, the integrin-mediated cell adhesion pathway emerged as a feature of chemo-resistant PDAC cells. ITGA5 forms a dimer with integrin beta-1, is responsible for adhesion to fibronectin, and is involved in the metastasis and invasion of malignant tumor cells [32]. The combined depletion of ITGA5 in PDAC cells as well as E- and P-selectins in host mice has been reported to significantly suppress intraperitoneal carcinomatosis in PDAC cells xenografted into immunodeficient mice [33]. Because chemo-resistant cells exhibited increased invasive ability compared with their parental cells (chemosensitive cells), the GSEA results regarding the integrin-mediated cell adhesion pathway demonstrated consistency. Thus, we considered integrin expression a chemoresistant cell phenotype.

In the initial microarray analysis of the 18 blood samples, miR-4658 showed the highest differential expression (S1 Table), and there were four other candidate miRNAs besides miR-26a-5p that we did not validate in this study. It remains unclear whether these miRNAs are related to PDAC prognosis or treatment response. Although reports on these miRNAs are limited, some studies have suggested a potential association with pancreatic cancer. Among these, miR-6835 has been reported to be associated with the presence of cancer and to decrease in resistant strains [34, 35], which aligns with our findings and is particularly intriguing, warranting further research. In our approach, we considered miR-6070, as it targets many ITGA-related proteins and was therefore initially included as a candidate in this study. However, due to the lack of reports on its association with pancreatic cancer, we chose to focus on miR-26a-5p.

In this study, miR-26a-5p expression was found to be a reliable biomarker. In other cancers, miR-26a-5p has been reported to act as a tumor suppressor. In hepatocellular carcinoma (HCC) and gastric cancer, miR-26a-5p expression has been reported to inhibit proliferation, migration, and invasion by downregulating oncogenic signaling pathways (e.g., the HGF-Met and COL10A1 pathways) [36, 37]. Moreover, a previous study reported that miR-26a governs tumor cell anoikis sensitivity by downregulating ITGA5 expression in HCC [31]. These findings corroborate those of our study.

We examined the relationship between miR-26a-5p expression and chemosensitivity. Growth inhibitory assays have revealed that miR-26a-5p inhibition results in lower chemosensitivity, and experiments on clinical specimens have demonstrated that significantly more patients in the high miR-26a-5p expression group complete adjuvant chemotherapy. The relationship between miR-26a-5p expression and the SAR was also consistent, and we considered miR-26a-5p a potential biomarker for the selection of postoperative adjuvant chemotherapy. In our validation of miR-26a-5p as a biomarker, we aimed to determine its ability to identify cases in which adjuvant chemotherapy would be effective. For this validation, we defined "effective adjuvant chemotherapy cases" as those in which patients did not experience recurrence during chemotherapy and remained recurrence-free for at least six months after completing chemotherapy. The sensitivity and specificity of miR-26a-5p expression for identifying these "effective cases" were 89% and 67%, respectively. However, it is important to note that the cases used for this validation were limited to those who experienced recurrence after adjuvant chemotherapy. Therefore, these sensitivity and specificity values may not fully reflect the real-world clinical scenario. Nonetheless, the results of this validation suggest the potential usefulness of miR-26a-5p as a biomarker in the adjuvant setting.

From the clinical results, high levels of miR-26a-5p were associated with low ITGA5 expression and seemed to correlate with effective adjuvant chemotherapy. To investigate the functional changes associated with these results, we performed assays to assess chemoresistance, invasion, and proliferation. The results indicated that high miR-26a-5p levels were associated with low ITGA5 expression, reduced invasion ability, and increased sensitivity to chemotherapy. However, proliferation ability was not affected. Conversely, when miR-26a-5p was knocked down, ITGA5 expression increased, invasion ability worsened, and chemoresistance became stronger. This suggests that reduced miR-26a-5p expression in subclinical tumors might be linked to increased invasion ability and chemoresistance, potentially making this miRNA a useful biomarker. However, the reasons for decreased miR-26a-5p expression remain unclear and require further investigation.

The integration of clinical blood sample data and cell line data was pivotal in identifying miR-26a-5p as a potential biomarker. MiRNAs extracted from patient blood reflect a complex interaction between the tumor, its microenvironment, and the body’s response. Analyzing blood samples allows us to account for inter-patient variability and differences in tumor characteristics. In contrast, using cell lines enables us to study gene expression changes in response to specific alterations in cellular characteristics.　By combining these approaches, we could distinguish between changes due to tumor-specific factors and those arising from patient-to-patient variability. This integrative approach was particularly valuable in narrowing down the list of potential biomarkers. Although it is currently unclear whether the other candidate miRNAs would have been useful, this integrative approach provided significant insights. Future research will involve exploring the role of other miRNAs not included in this study to further validate and potentially discover additional biomarkers.

The present study has certain limitations. The relationship between chemosensitivity and proteins associated with invasive ability remains unclear. Although epithelial mesenchymal transition is one of the keywords in this issue, further experiments are required to elucidate these relationships. Moreover, the clinical samples were limited, and further validation is required to support this hypothesis.

## Conclusions

Our study provides a novel and reliable biomarker. The expression of miR-26a-5p in postoperative blood is a biomarker that indicates the presence of subclinical tumors and their chemo-susceptibility. If patients exhibit high miR-26a-5p expression in the blood after radical resection for PDAC, prolonged adjuvant chemotherapy may improve their prognosis.

## Supporting information

S1 Fig Relationship between integrin proteins and miR-26a-5p expression in PDAC cells. **(A-B)** ITGA6 and ITGA8 expression levels were assessed by qRT-PCR in MiaPaCa2, BxPC3, and MiaPaCa2-GR10 (GR10) cells. *P < 0.05; **P < 0.01; ***P < 0.001.
(PDF)

S1 Table Results of microRNA array from 18 cases. (XLSX)
