# Supplementary figures and images for: MicroRNA-26a-5p is a reliable biomarker in the adjuvant setting for pancreatic ductal adenocarcinoma
Source: PLoS One. 2024 Sep 17;19(9):e0310328. doi: 10.1371/journal.pone.0310328 (PMC11407630; doi:10.1371/journal.pone.0310328)

S1 figure

**A**

ITGA6

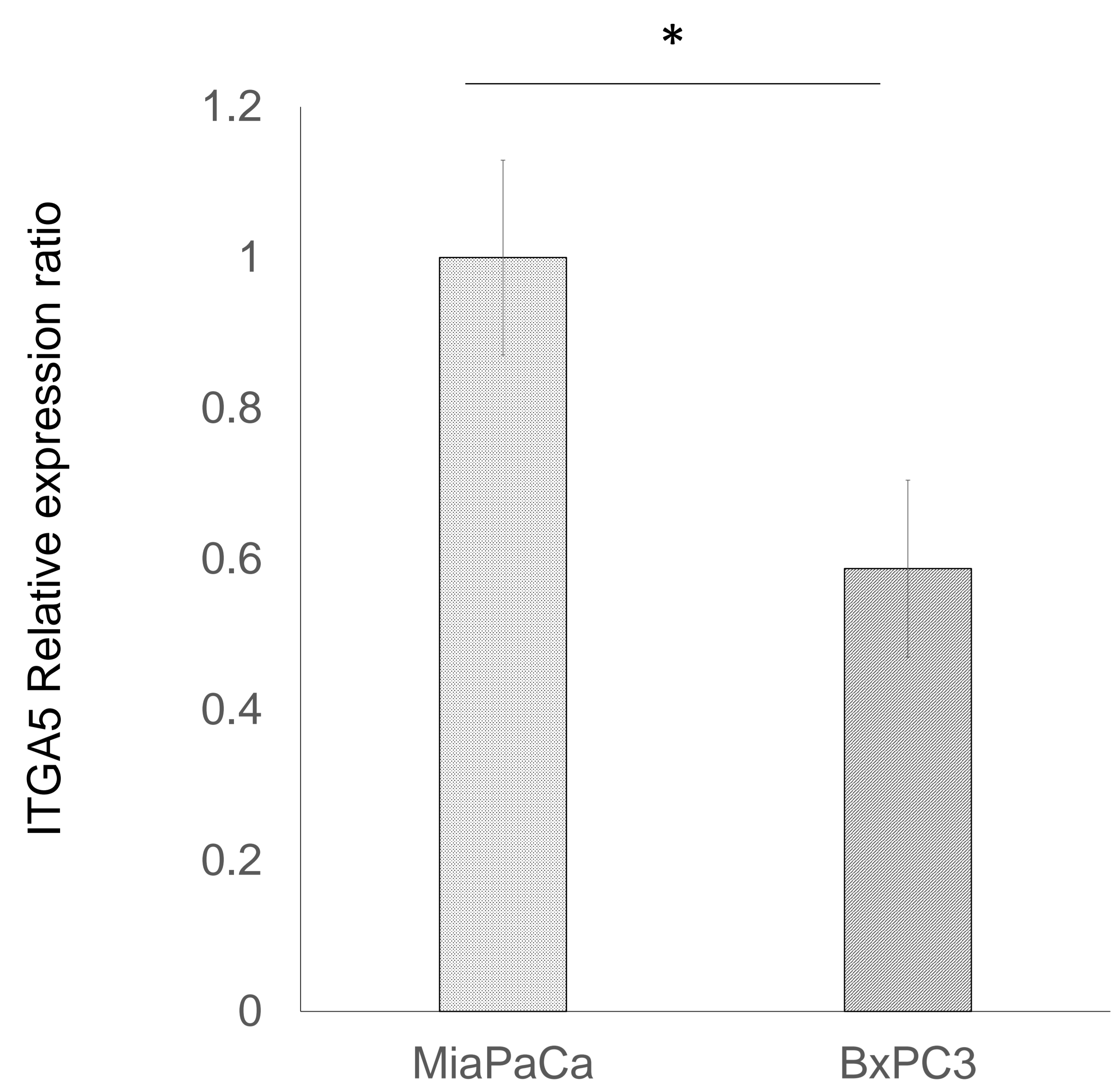

ITGA6

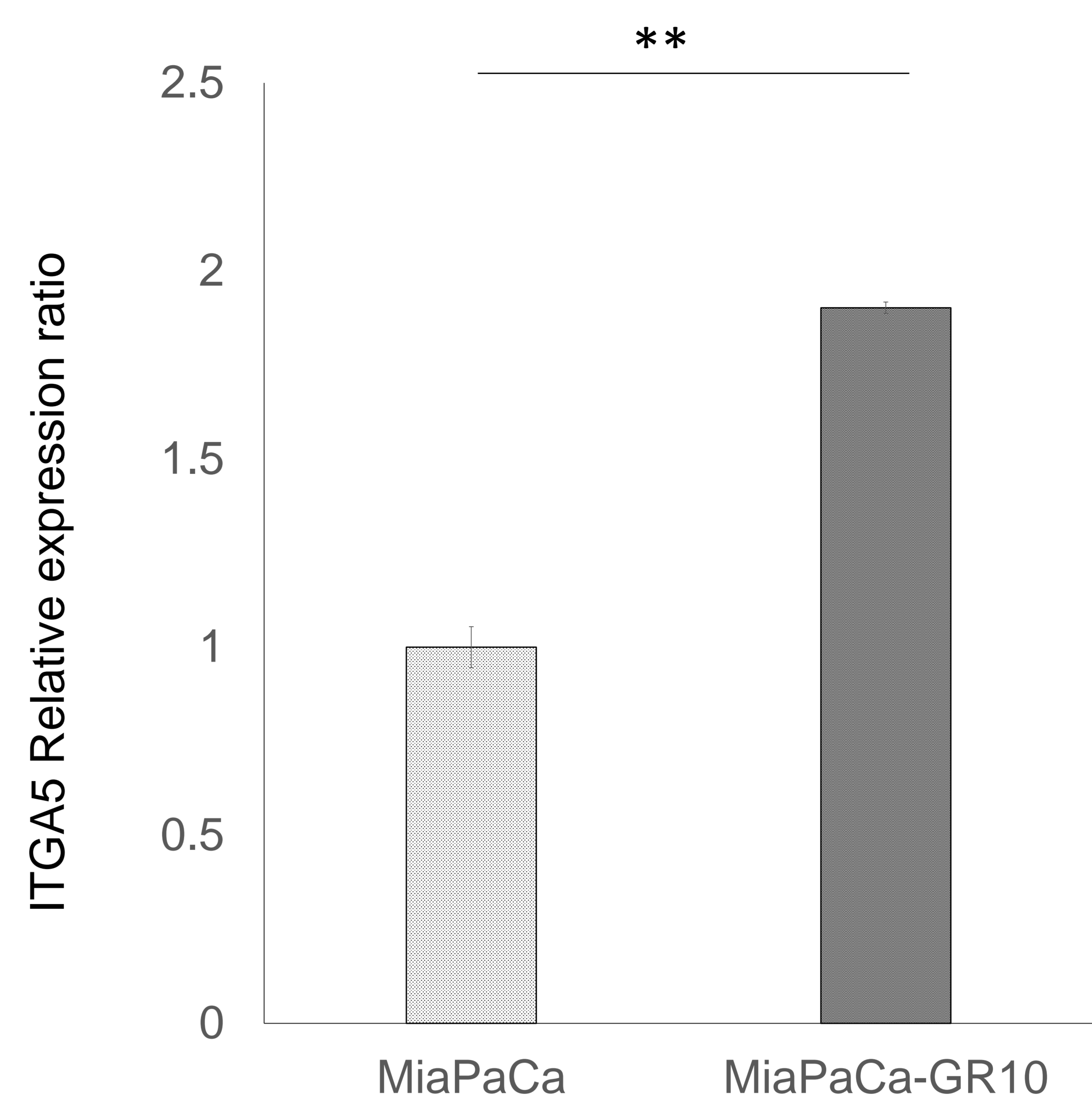

**B**

ITGB8

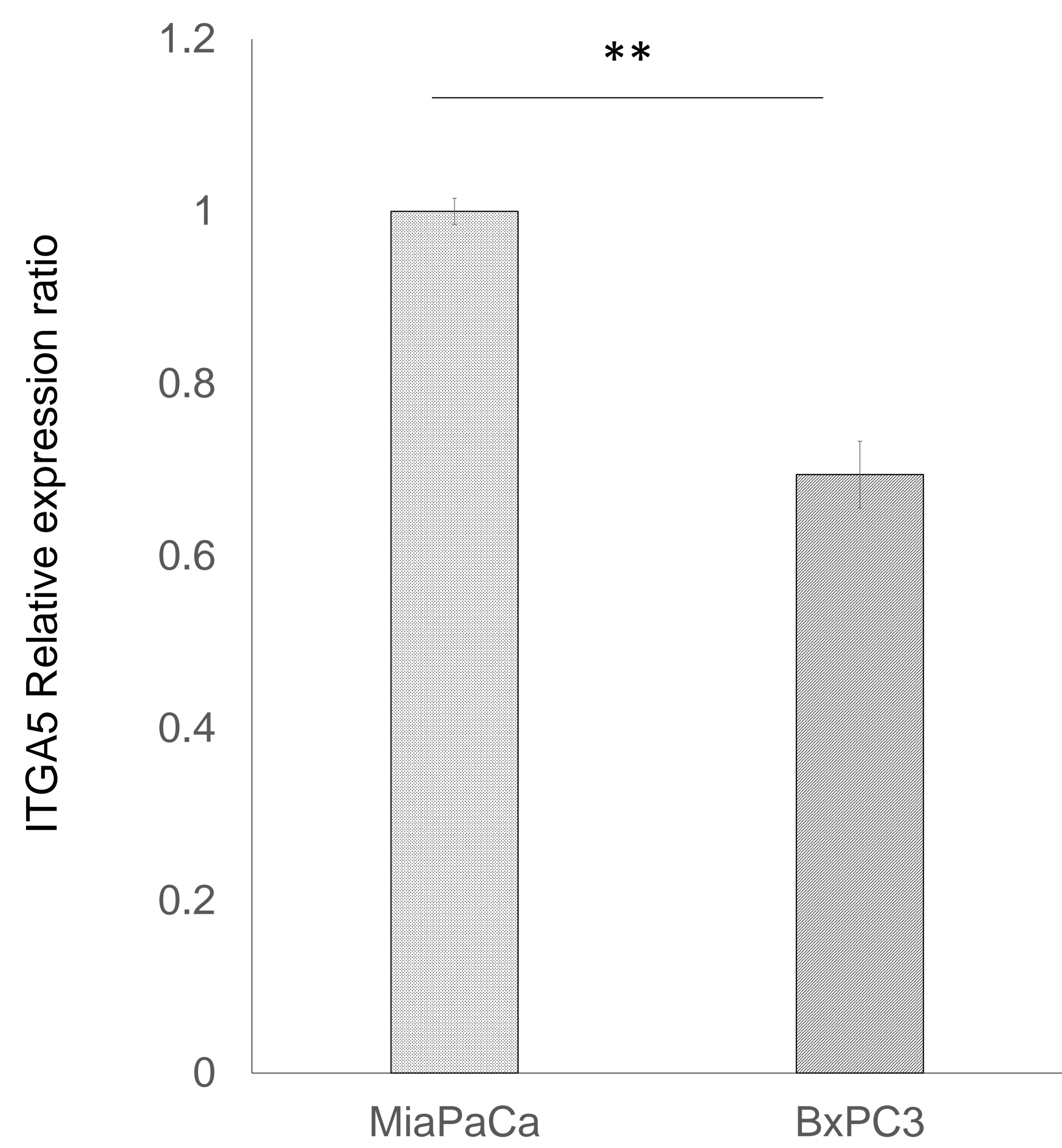

ITGB8

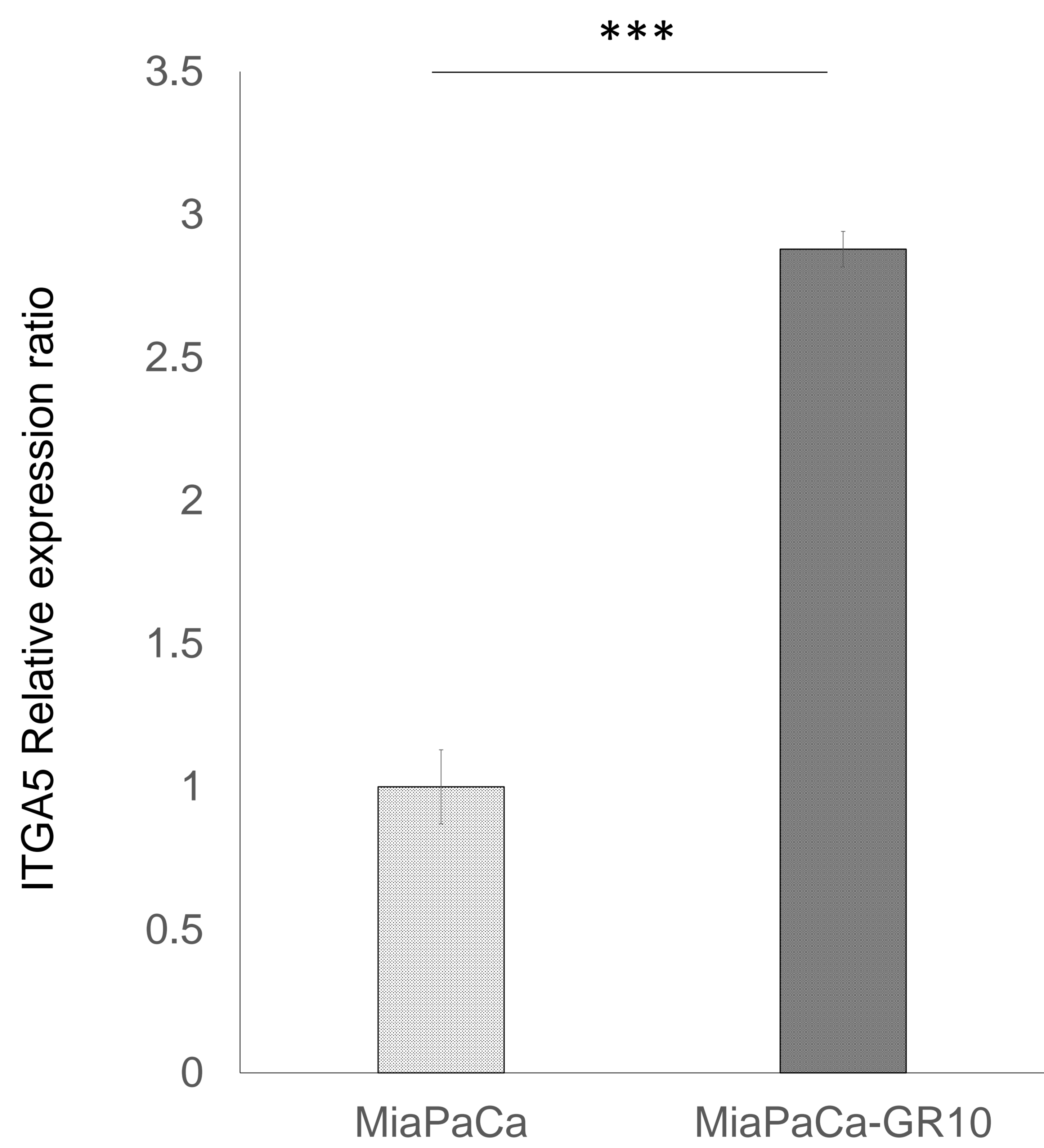

Supplement: S1 Fig — (A-B) ITGA6 and ITGA8 expression levels were assessed by qRT-PCR in MiaPaCa2, BxPC3, and MiaPaCa2-GR10 (GR10) cells. *P < 0.05; **P < 0.01; ***P < 0.001. (PDF) [file pone.0310328.s001.pdf]
